# Evaluation of a Residential Mental Health Recovery Service in North Queensland

**DOI:** 10.3389/fpubh.2018.00123

**Published:** 2018-05-02

**Authors:** Marion Heyeres, Irina Kinchin, Elise Whatley, Lisa Brophy, Jon Jago, Thomas Wintzloff, Steve Morton, Vinitta Mosby, Narayan Gopalkrishnan, Komla Tsey

**Affiliations:** ^1^College of Arts, Society and Education, James Cook University, Cairns, QLD, Australia; ^2^Centre for Indigenous Health Equity Research, School of Health, Medical and Applied Sciences, Central Queensland University, Brisbane, QLD, Australia; ^3^The Cairns Institute, James Cook University, Cairns, QLD, Australia; ^4^Mind Australia Ltd., Heidelberg, VIC, Australia; ^5^Centre for Mental Health, Melbourne School of Population and Global Health, The University of Melbourne, Melbourne, VIC, Australia

**Keywords:** mental health, mental illness, service, recovery, community, subacute, step-up step-down

## Abstract

**Background:**

Evidence shows that subacute mental health recovery occurs best when a person remains active within the community and fulfils meaningful and satisfying roles of their choosing. Several residential care services that incorporate these values have been established in Australia and overseas.

**Aims:**

This study describes (a) the development of an evaluation framework for a new subacute residential mental health recovery service in regional Australia and (b) reports on the formative evaluation outcomes.

**Methods:**

Continuous quality improvement and participatory research approaches informed all stages of the development of the evaluation framework. A program logic was established and subsequently tested for practicability. The resultant logic utilizes the Scottish Recovery Indicator 2 (SRI 2) service development tool, Individual Recovery Plans (IRPs), and the impact assessment of the service on psychiatric inpatient admissions (reported separately).

**Results:**

Service strengths included a recovery-focused practice that identifies and addresses the basic needs of residents (consumers). The consumers of the service were encouraged to develop their own goals and self-manage their recovery plans. The staff of the service were identified as working effectively in the context of the recovery process; the staff were seen as supported and valued. Areas for improvement included more opportunities for self-management for residents and more feedback from residents and carers.

## Introduction

Globally, mental illness accounts for 7.4% of the world’s measurable burden of disease (1). According to the 2014–2015 National Health Survey, an estimated four million people living in Australia, equating to 17.5% of the population, experienced mental and behavioral conditions (2). The most prevalent mental disorders are depression, anxiety, alcohol abuse, and personality disorders (3). Each year almost 64,000 Australians aged 18–64 who are affected by psychotic illness and are users of public specialized mental health services are severely ill and have complex needs (4). Mental illness can have severe effects on the individuals and families concerned and may contribute to poverty, unemployment, and homelessness. Broader societal impacts affect high unemployment, with Australia showing one of the lowest rates of employment participation by people with lived experience of mental illness (5). Furthermore, the annual cost of mental illness in Australia has been estimated by the Australian Bureau of Statistics at $20 billion, which includes the cost of loss of productivity and labor force participation (6). The cost of the burden of severe mental illness, including opioid dependence in Australia in 2014, was estimated over $99 billion (7).

A key feature of the deinstitutionalization of people with serious mental illness has been to reduce the use of large, stand-alone, long-stay institutions and alternatively offer shorter stay inpatient services, often attached to large health services. However, psychiatric inpatient care has often been experienced as non-therapeutic, overcrowded, inefficient, and poorly organized, leading to high levels of stress for staff and a poor patient experience (8).

The challenges of people with mental health conditions led to the recovery movement emerging in the late 1970s, which has subsequently impacted mental health policy and practice in the United States, United Kingdom, Australia, and New Zealand (9). According to Davidson (9) “this movement emphasized lessons learned from the life experiences of these people as they found their way often without assistance from, and in some cases despite, mental health services to leading “meaningful and productive” lives in their communities” (p. 1). In support of this claim, longitudinal data also show that people with severe mental illness can eventually recover, given the right circumstances (9). However, it is important to understand the term “recovery” in the context of the mental health recovery movement. Recovery is a deeply personal, and unique process, and is not about the presence or absence of symptoms (10), but the restoration of functioning for individuals, above and beyond the reduction of symptoms (9). People affected by mental health challenges have increasingly conveyed that what is needed for their recovery is support to live independently (10).

Saunders et al. (11) emphasize ideas of hope and meaning being at the core of recovery for people with mental health conditions, and therefore enabling them to move onto a satisfying life even within the limitations caused by illness (10). Hence, the shift toward community-based recovery care services emerged, as health-care providers acknowledged the organizational, economic, and health-related benefits of recovery of people who require an acute residential level of care (12). Evidence shows that recovery occurs best when a person remains active within the community and fulfils meaningful and satisfying roles of their choosing (13). Several residential care services that incorporate these values and actively involve family and community have been established in Australia and overseas (14). In addition to the provision of crisis care, these services tend to also cover support with housing, day to day activities, social and financial skills, community access, social and recreational activities, counseling and advocacy, and vocational and employment support (13).

Between 2014 and 2015, about 7,750 episodes of residential care for around 5,800 people were recorded in Australia (15). This equates to an average of 1.3 episodes of care per resident and 39 residential care days per episode. These figures related to all episodes of care in all government-funded residential mental health services in Australia and included rehabilitation, treatment, or extended care. The typical length of stay for a completed residential care episode was 2 weeks or less, with 1.9% of them lasting longer than 1 year (15). One of these residential services is the Adult Prevention And Recovery Care (PARC) service which on the service level continuum sits between adult psychiatric inpatient units and comprehensive community treatment provided in a consumer’s usual place of residence, sometimes described as “subacute” (16). PARC services involve a partnership between community-managed mental health support services (i.e., non-government mental health agencies) and clinical mental health service providers. They have a strong emphasis on integrating clinical mental health care with intensive recovery-focused psychosocial input. The pathway to admission can be described as a step-up from the person’s place of residence or a step-down from a psychiatric inpatient unit (17). With the provision of an alternative to inpatient treatment, PARC aims to minimize the risk of symptom intensification, have a positive impact on the pattern of illness, and to reduce the adverse consequences for individuals, their families, and carers. Admission to the PARC service is voluntary and for a maximum stay of 28 days. Consumers with increasing symptom severity may enter as a step-up from community care, to avoid hospital admission. Inpatients from psychiatric units, who still need a residential level of care, can step-down to the PARC facility, hence potentially reducing their hospital length of stay (17). There are early signs that PARC services and their equivalents are well regarded by those they serve (particularly when they are staffed by supportive and caring workers and offer practical assistance, therapeutic activities, and socialization opportunities) and are associated with improvements in recovery-related indicators (e.g., role functioning) and symptom-based measures. However, the evidence base is limited (18–21).

As a result of population growth in Far North Queensland, Cairns and Hinterland Hospital and Health Service (CHHHS) found it difficult to manage the increased numbers of people requiring psychiatric inpatient and subacute care (22, 23). In response to this increased demand for the service, Mind Australia, in partnership with CHHHS, opened a PARC service in Cairns in May 2015. Although consumers have to meet certain criteria to fulfill eligibility for admission, Cairns PARC is a voluntary service. During the first 3 days of entry to the Cairns PARC, PARC staff offer orientation sessions to introduce and welcome the consumer. In collaboration with the consumer, a formal Individual Recovery Plan (IRP) is prepared. This plan comprehensively assesses the needs of the consumer, estimates the person’s length of stay, the type of issues that need to be addressed, and develops active strategies for discharge. Consumers are actively involved in creating individual recovery care, crisis, and relapse prevention strategies from day 1 at PARC.

Daily PARC routine includes a mixture of structured activity and individual one-on-one support time. Accommodation and meals are provided free of charge, and in turn, consumers are expected to contribute to the running of the facility, including assisting with the cooking of shared meals and cleaning. Families and carers may be involved in care planning and provided psychoeducation and referral to community supports. Assistance may be provided to link with community services such as clubhouses, employment services, and mental health. The proposed discharge time frame identified at the commencement of care is regularly reviewed with the consumer. Once the discharge criteria are fulfilled or defined, and IRP goals and objectives and agreed timeframes have been achieved, the consumer is ready to exit the service. In some cases, where there are concerns about the consumer’s mental health, the consumer can be transferred to the acute unit for further treatment and support. PARC discharge pathways can be with or without outreach support.

Soon after the establishment of PARC Cairns, Mind Australia Ltd. contacted researchers from James Cook and Central Queensland Universities (JCU and CQU) seeking to evaluate the early days of the service and to develop an evaluation framework that can demonstrate the effectiveness of the service over an extended period. This study aims to describe the development and application of this evaluation framework that provided useful learning from the Cairns PARC first year of operation. The team of PARC staff, university researchers, and stakeholders discussed the appropriate terminology to refer to people with mental health conditions and decided on the term “consumers,” which is applied consistently throughout this study.

## Methods

### Developing an Evaluation Framework

Continuous quality improvement and participatory research approach informed all stages of the development of the evaluation framework (24). Initially, the six-stage evaluation model by Dehar et al. (25) was utilized: 1—engagement with stakeholders; 2—service description; 3—evaluation framework; 4—data collection; 5—justification of conclusions, and 6—ensure continuous use, and share lessons learned. The model was then monitored and evaluated to determine the extent to which it worked in practice, using two different tools: The Scottish Recovery Indicator 2 (SRI 2) (26) and customized IRPs with a scoring scale. Descriptive analysis was applied to the quantitative data of the Scottish Recovery Indicator 2 (SRI 2) (27), and thematic analysis to the qualitative data of the IRPs (28).

After completion of stakeholder engagement, an evaluation logic model was established (Figure S1 in Supplementary Material). Such a model not only depicts how a program is supposed to work, it first and foremost offers a systematic approach to the program planning, implementation, and evaluation (29). In PARC’s case, the model started by reflecting on five core principles of recovery: collaboration, least possible restrictive practices, respect and responding to diversity, consumer and carer participation, and privacy and confidentiality (17). Then, it described the activities that were provided by the service, followed by a description of intended outcomes. By clarifying activities and intended outcomes through discussion in the developmental stage of the model, the change processes underlying a program become visible.

The logic model describes the goals of the service, assumptions underpinning the model, and activities that needed to be in place to achieve the goals. The support of personal recovery and well-being was delivered by a mix of clinical and psychosocial treatments, as well as rehabilitation support, education, and training, provided in a minimally restricted environment. The service was committed to evidence-based practice underpinned by trust, sensitivity, and partnership. Established partnerships with the health and education sector, communities, and organizations were sought to be sustained long term. PARC’s recovery-oriented culture was based on a service model that reflected service values and guidelines, delivered through daily routines and actions. Daily psychological recovery review was undertaken in collaboration between consumers, clinicians, and key workers. A Family Engagement Worker was available to work with more complex cases. To ensure the practice was needs-sensitive, consumers and their carers were invited to participate actively in the service planning. Furthermore, it was imperative that staff reflected the cultural diversity of consumers and committed to the continuous quality improvement of the service. The model also served as a guide for the data collection process and enabled stakeholders to clarify the service’s strategies; therefore, contributing to improved and focused service.

### Data Collection Tools

The data collection process was a collaborative effort with clearly defined roles from the beginning. The PARC service was responsible for IRPs data collection. University researchers analyzed deidentified IRPs data. One of the authors (LB) facilitated data collection and facilitated the data collection and analysis using the Scottish Recovery Indicator 2 (SRI 2) tool. Mind Australia routinely collects SRI 2 data as part of their quality assurance activities. Once these steps were completed, both parties jointly interpreted the results.

### The Scottish Recovery Indicator 2 (SRI 2) Tool

The Scottish Recovery Indicator 2 (SRI 2) tool (26) was selected to determine whether the service met its objectives as viewed by staff members, those using the service, and their families and carers. The SRI 2 framework included a series of reflective statements that allow services to rate current practice against the 10 recovery indicators. These ratings are intended to stimulate thoughtful conversations about current strengths and potential areas for improvement. The SRI 2 was administered first in 2015 and repeated in 2016. The main outcome from the SRI 2 process was an action plan. This plan recorded existing strengths with a potential to be built upon, and improvements that could make the service more recovery focused (30).

Based on robust evidence about what works to support recovery, the 10 recovery indicators used in this evaluation were as follows: 1. Basic needs are identified and addressed; 2. Goals are identified and addressed; 3. Personalized services are provided; 4. Service is strength based; 5. Service promotes social inclusion; 6. Service promotes and acts on service user involvement; 7. Informal carers are routinely involved; 8. Service encourages advance planning and self-management; 9. Staff are supported and valued; and 10. Practice is recovery focused. Over 2 days, nine PARC staff participated in group discussions to review service information, assessments and recovery plans, and to reflect on service provider (staff) views. Data from service users, consumers, and informal carers were collected between August and December 2016. Data sheets were completed by 5 service provider staff, 14 service users, and 1 informal carer. The data were analyzed using descriptive analysis.

### Individual Recovery Plans

To supplement the SRI 2, the 3-month period from July to September 2016 was randomly selected to collect data from the IRPs, which are established on admission into the service. With the support of service staff, consumers were encouraged to record their goals, and action steps on how to achieve them. The plans also recorded data on support persons nominated by the consumer, a review date, and a 5-point Likert scale to rate their progress. Thematic analysis was applied to the qualitative data of the IRPs (28).

### Ethics

This study is a quality assurance activity and was granted an exemption from ethical clearance by the Far North Queensland Human Research Ethics Committee (HREC/15/QCH/108—1006 QA).

## Results

In alignment with the evaluation framework, two data sets provided first insights into short-term service delivery outcomes. The SRI 2 highlighted service strengths and areas for improvements, while data from the IRPs gave insight into the range and types of recovery goals and activities consumers reported.

### Scottish Recovery Indicator 2 (SRI 2)

Table 1 provides a comparison of the numbers of participants in the 2015 and 2016 SRI 2 evaluation process. Almost twice as many service users participated in 2016 (*n* = 14), compared with those in 2015 (*n* = 8). Only one (*n* = 1) informal carer participated in 2016, while nine (*n* = 9) participated in 2015. The number of service providers’ participation was similar in both years, 10 (*n* = 10) in 2015 and 9 (*n* = 9) in 2016. Due to low participation of informal carers in 2016, the results in this category are not representative of all informal carers.

**Table 1:** Cairns Prevention And Recovery Care (PARC) evaluation participants in 2015 and 2016.

Participants	2016	2015
Service users (consumers)	14^a^	8
Informal carers (families and carers)	1	9
Service providers (Cairns PARC staff)	9^b^	10

*^a^Participants included outreach consumers*.

*^b^Although 9 team members participated in the SRI 2 discussions, 5 team members completed Service Provider data collection sheets. Seven team members participated on day 1; however, 2 team members had to leave before the Service Provider data collection sheets being completed*.

Each recovery indicator was used to measure a range of areas of practice, including strength-based assessments (SBAs), recovery plans, service information, service providers, service users, and informal carers. Table 2 provides a summary of results including average scores for each recovery indicator, and overall average scores. For each type of data, participants rated the extent to which they agreed or disagreed with each of the statements on a scale of 1–5; 1 = strongly disagree and 5 = strongly agree. The overall results were highlighted *via* a traffic light system. Green indicated a high score, orange indicated a medium to high score and, and red indicated room for improvement. The majority of overall average scores are the same or higher than in 2015; therefore, indicator scores that may have been orange in the 2015 report may be green in 2016, because overall service improvement appeared to occur between SRI 2 reviews.

**Table 2:** The Scottish Recovery Indicator 2 (SRI 2) data 2015 versus 2016.^a^

Indicator/statement	Data type (average score)												Overall average score	
	Assessment		Recovery plan		Service information		Service provider		Service users		Informal carers			
						
	2016	2015	2016	2015	2016	2015	2016	2015	2016	2015	2016	2015	2016	2015
	
	*n* = 10	*n* = 1	*n* = 10	*n* = 1	N/A	N/A	*n* = 5	*n* = 10	*n* = 14	*n* = 8	*n* = 1	*n* = 9		
Basic needs are identified and addressed	4.5	5.0	4.7	4.0	N/A	N/A	4.6	4.1	4.4	4.5	5.0	3.9	4.6	4.3
Goals are identified and addressed	4.3	4.0	4.4	5.0	N/A	N/A	4.6	4.4	4.4	4.1	N/A	N/A	4.4	4.4
Personalized services are provided	4.5	5.0	4.9	5.0	3.0	3.0	4.6	4.0	4.4	4.4	N/A	N/A	4.3	4.3
Service is strengths based	3.9	4.0	3.4	2.0	4.0	2.0	4.3	4.2	4.1	4.4	N/A	N/A	3.9	3.3
Service promotes social inclusion	4.0	4.0	4.0	2.0	4.0	2.0	4.2	3.3	4.1	4.3	N/A	N/A	4.1	3.1
Service promotes and acts on service user involvement	N/A	N/A	N/A	N/A	4.0	2.0	4.2	3.2	4.1	4.4	N/A	N/A	4.1	3.2
Informal carers are involved	3.6	2.0	2.5	2.0	5.0	2.0	4.0	3.8	4.2	4.4	4.0	4.1	3.9	3.1
Service encourages advance planning and self-management	3.7	5.0	3.7	3.0	3.0	2.0	4.2	3.7	4.1	4.3	4.0	4.1	3.8	3.7
Staff are supported and valued	N/A		N/A		5.0	2.0	4.2	3.9	4.3	4.6	4.0	4.7	4.4	3.8
Practice is recovery focused	4.5	4.0	4.1	5.0	5.0	3.0	4.8	4.3	4.6	5.0	5.0	4.3	4.7	4.3
Overall average score	4.1	4.1	4.0	3.5	4.1	2.3	4.4	3.9	4.3	4.4	4.4	4.2	4.2	3.7

*^a^A traffic light system has been used to show changes in average scores. Green indicates that the average score has increased by ≥0.2; orange indicates that the average score has stayed the same or similar (≤0.1 difference); and red indicates the score has decreased by ≥0.2. It should be noted that changes to the way assessment and care plan data were gathered may explain some variability in results between 2015 and 2016. Traffic lights have not been added for informal carers due to the low number of participants in 2016 (*n* refers to the number of data collection sheets for each data type)*.

Overall, results indicate that PARC Cairns is running a strong recovery care service. PARC staff and service provider feedback regarding “staff being supported and valued” indicated a very positive result and was justified by a large amount of policy and procedure available on the Mind intranet about staff training, supervision and well-being. By contrast, the scores of services users on the same statement indicate a loss of 0.3 points (as above), with a still fairly high individual score of 4.3 out of 5. Other positive results about PARC staff indicate that they are well regarded among consumers and informal carers due to their friendly, considerate, and approachable demeanor, with their practical approach to involving consumers in real life tasks. As an example, one consumer remarked “I found if they had not spotted me during the day, there was always that knock on my door to see how I was going which was great. Even with my condition, after a few days, I felt comfortable enough to approach staff. They are all friendly, considerate, and helpful in any way they can.”

Intense focus on recovery care was another highlighted strength, with an average score increase by 0.4, to 4.7 points. The participants rated “practice is recovery focused” highly, as a number of assessments “looked at hopes and plans for the future,” reflected on “positive outcomes” and used hopeful and future-focused language. Furthermore, PARC and Mind Service brochures explicitly discussed recovery and how consumers could use PARC and other Mind services for their recovery. One carer commented “I consider PARC to be a vital step for people on their touring to a better life. Their stay at PARC gives them a clean, supportive, environment to set some life goals, and time out to gain some perspective.”

The team rated “basic needs … ” highly because across the range of assessments there was evidence of discussion with clients in each of the domains (life areas) in the SBAs. On arrival, consumers were welcomed and shown around the premises before meeting with staff to identify the goals they intended to work toward during their stay. IRPs reflected important issues that were identified as priorities by clients such as health, housing, jobs, social connection, finance, and healthy eating. Through brainstorming activities, the consumer was then supported to come up with manageable strategies to achieve their goals. This information was recorded on IRPs and revisited regularly to enhance recovery. Although receiving a strong overall score, there was a decrease in the average score for “goals are identified and addressed” in the IRPs from 5 in 2015 to 4.4 in 2016. This decrease could be attributed to staff being asked this year to rate goals on whether they used an SMART approach (Specific, Measurable, Achievable, Relevant, and Time-Limited). Staff reflected that a proportion of the plans reviewed did not include timelines.

Looking into individual data sources of the 2016 scores, such as assessments and recovery plans, some of the areas that regressed were basic needs (−0.5); goals (−0.5); personalized services (−0.5); advance planning and self-management (−1.3); and recovery-focused practice (−0.9). Service users provided lower scores in the areas of strength-based service (−0.3); social inclusion (−0.2); user involvement (−0.3); informal carers involved (−0.2); advance planning and self-management (−0.2); staff feeling supported and valued (−0.3); and recovery-focused practice (−0.4). PARC Cairns acknowledged the issues that transpired from the evaluation results and brainstormed action steps to address them. Some of the areas where PARC could improve on their service included better quality and processes of their SBA and IRPs; more opportunities for self-management for residents; better focus on self-management in service information; and more feedback from residents and carers.

Looking at the IRPs, while there was the use of strengths-based language and mention of strengths in some of them, other plans did not refer to strengths or did not appear to “mobilize” strengths that clients had mentioned in their SBA. Giving consumers the opportunity to self-manage had been addressed for a few, however, not all IRPs offered detailed descriptions of, who was involved. In acknowledging these areas where there is “room for improvement,” PARC recorded the following action steps: increasing the use of verbal and written strength-based language; inviting consumer self-determination, perspective and language on IRPs, and update and share these plans more regularly; engaging families and carers in feedback processes; and upskilling staff in motivational interviewing to better support consumers in goal setting.

### Individual Recovery Plans

Over the three months period (from July to September 2016), 45 consumers were admitted to PARC; of those, 22 were females. Of the 45 (*n* = 45) consumers admitted over the 3 months, 22 (*n* = 22) were female, 21 (*n* = 21) were male, and 2 (*n* = 2) missing values. The average duration of stay per consumer was 15 days. The shortest stay was 1 day (*n* = 2), and the longest was 32 days (*n* = 1). Of the 45 (*n* = 45) consumers admitted, IRPs were on file for 44 (*n* = 44), and 1 (*n* = 1) was missing. At discharge, 12 (*n* = 12) consumers rated their progress, 28 (*n* = 28) did not rate their progress, and 5 (*n* = 5) were either unable or refused to do so. Of the 12 (*n* = 12) consumers who rated their progress, 6 (*n* = 6) were males, 5 (*n* = 5) were females, and 1 (*n* = 1) did not identify gender. PARC staff acknowledged that the chosen 3-month period was particularly out of character regarding the completion of IRP documents. The low response rate to consumer progress (27%) was attributed to premature discharge, mental or emotional incapacity, or other personal reasons. Given the fact that this was a pilot study, the key characteristics and themes identified from the 12 completed IRPs will inform a most likely significant revision of this part of the evaluation.

The 12 (*n* = 12) consumers who rated their progress and achievements, recorded a total of 31 (*n* = 31) goals. Thirteen (43%) were rated as completed, and one (3%) as almost completed. Eight (26%) were rated as some progress being made, and two (6%) between some and no progress. Five (16%) goals were rated as no progress. Two (6%) goals were not rated. It must be noted that the rating reflected perceived progress, dependent on various stages of personal recovery and may not be representative of the quality of service provided. Also, length of stay impacted on the completion of rating, as well as the level of attainment achieved. For example, the goals identified at the commencement of care were typically revisited after 14 days. If the consumer exited the service before that, depending on the reasons, recovery plan progress rating might not have been a priority. Furthermore, a short duration of stay may not have given enough time to generate any significant progress, and therefore a rating seemed unwarranted to the consumer. As described earlier, data collected from the IRPs served to identify gender, the length of stay, type of goals, strategies to achieve these goals, support person involved, and rating of progress.

Recorded goals fell into the areas of health, finances, education, employment, housing, life skills, relationships, and other (Table 3). The most frequently mentioned goals pertained to the consumer’s physical, mental, and dental health. Strategies to achieve goals in these areas ranged from making health service provider appointments; planning, preparing, and consuming healthy meals; physical exercise; and to having medication reviewed. In the category of life skills, consumers reported the wish to improve their social interaction and to have meaningful relationships. Strategies to achieve these goals ranged from increasing participation in activities and socializing with co-residents and staff. An equally important goal centered around relationships and family. Consumers wished to have more or regular contact with their partner or children. They intended to achieve these goals by asking for help with making phone calls, sending mail, and arranging meetings. Regarding finding suitable housing on discharge, consumers recorded registering with agents, completing required documentation, and considering shared/family accommodation. Furthermore, consumers considered contacting Lifeline or the Salvation Army to obtain used furniture or look for already furnished apartments. The thought of going to work, or back to work, seemed to cause consumers a considerable amount of anxiety. Strategies to overcome their anxiety were learning stress management techniques and visiting a psychologist.

**Table 3:** Characteristics of IRP.

ID	Gender, M/F	Length of stay (days)	Individual recovery goals	Strategies to achieve the goals	Person who filled IRP	Rating (1–5)^a^	Notes
1	F	14	Obtain optimal health	Eating well, preparation of meals, look for recipes	Self/staff	5	
			Get back to work	Manage stress and anxiety; less stress management techniques; see a psychologist	Self/staff	3	
2	Not reported	14	Social interaction	Join groups; socialize with co-residents and staff	Self/staff	5	
3	M	11	Find accommodation	Visit housing hub; apply for accommodation	Self	1	Change of circumstances; mother moved to Cairns and consumer moved in with her
			Medications	Medication review	Staff	5	
4	F	15	Cooking	Go to the kitchen and assist	Self/staff	2	
			Physical health	Set plan for the day; go for a walk around lunchtime	Self/staff	3	
			Learn about nutrition	Google things; worker to download sheets	Self/staff	3	Learned from evening cooking
5	F	14	Find suitable accommodation	Approach real estate agents	Self/staff	5	
			Sort out belongings, personal comforts, etc.	Shopping	Self/staff	5	
			Counseling	Get domestic violence counseling	Self/staff	5	
			Contact with children	Receive phone calls; send mail	Staff	5	
6	M	28	Obtain ID and access money	Ring bank; Medicare; health care	Self/staff	4	Sorted out bank and got Medicare card
			Obtain accommodation	Real estate, shared accommodation	Self/staff	N/A	Looking into it this week
			Dentist	Ring to make appointment	Self/staff	N/A	Looking into it Monday
7	M	14	Social interaction	Participate in some of the group	Self/staff	3	
			Visits from family, wife, grandson	Ask to use office phone; arrange visits	Self/staff	1	One visit from grandson
8	F	13	Social interaction and establish meaningful relationships	Join hobby groups and Prevention And Recovery Care (PARC) outings	Self/family	5	
			Medication stability	Make use of support systems and people	Self/staff	5	
9	F	14	Find suitable accommodation	Fill out rental application; search real estate.com	Self	5	
			Get furniture or furnished unit	Contact Salvo and Lifeline	Self/staff	1	
			Seek regular access to children	Set regular times and maintain phone calls.	Self	5	
10	M	21	Get teeth fixed	Make dentist appointment	Staff	3	X-ray done, waiting on list
			Finances	Get on disability pension	Self/staff	5	
			Exit into accommodation	Contact homeless hub; register with housing; moving in with brother	Self/staff	5	Moved in with brother
11	M	22	Give up all bad things	Be rehabilitated; cut off all negative people; getting involved in …?	Self/staff/family	3	
			Get a job	Got to NEATO (employment agency) and speak to neighbors	Self	1	
			Get life organized—study	Study next year a certificate in mental health work and support work	Self/staff	1	
12	M	32	Improve mental health	Getting sorted out with Clozaril; interact with PARC staff	Self/staff/medical	3	Still on Clozaril startup; has been interacting with staff and co-residents
			Independently cook when moving out	Help out with PARC dinner group preparation; cook lunch with key worker	Self/staff	3	Helped with group dinner and cleaning up; independently making lunches
			Keep accommodation clean and tidy	Stay on top of cleaning room	Self/staff	2	Cleaned unit with staff reminders

*M/F, male/female; IRP, Individual Recovery Plan*.

*^a^IRP ratings scale: 1 = no progress; 3 = some progress; and 5 = completed*.

On the more practical side, consumers recorded ideas of speaking to neighbors, or an employment agency, to find work. In the life skills category, consumers referred to hygiene, nutrition, and cooking skills. To keep their accommodation clean and tidy, they recorded the need to stay on top of their cleaning routine. Others were keen to learn about nutrition and planned to search Google to download relevant information. To be able to cook meals for themselves once discharged, consumers were keen on helping prepare meals. To get finances under control, one consumer intended to apply for the disability pension by visiting Centrelink. Another consumer recorded the intention to commence studies for a certificate in mental health and support work in the following year. Remaining goals recorded covered several areas of life, for example, obtaining a personal ID to arrange bank and health-care access. One consumer recorded their goal to be giving up all bad things, cutting off all negative people and being rehabilitated.

## Discussion

The PARC evaluation framework was developed to explicate the service delivery model and clarify the context in which the service was developed, the rationale behind the recovery approach, the activities designed to support and enable consumers to work towards their recovery goals and aspirations. The framework supports the quality improvement measures and enables staff, consumers, and carers to track and evaluate the service outcomes collaboratively.

Results from the analysis of the SRI 2 indicated that PARC’s focus on recovery care was a significant strength of the service and integrated well with consumer goals that were reflected in the IRPs. Furthermore, PARC staff were well regarded among consumers and their carers, especially in the context of their practical approach to involving consumers in real life tasks. PARC staff were particularly well skilled in helping the consumer defining their goals, and to maintain and improve this skill, the in-service “Mind Recovery College” was created. This unique approach was designed to support staff, stakeholders, and anybody from the public to learn more about mental illness, with the subject content being delivered by “teachers” with lived experience. The college developed a toolkit that supports consumers in achieving their goals. In relation to staff well-being and support, staff indicated that a wide range of policies and procedures were available to them on the Mind intranet about training, supervision, and well-being. Consumer feedback supported the SRI 2 indicator on staff feeling supported and valued, for example, one consumer commented that staff seemed satisfied in their work as “… they are always cheerful and obliging.”

Typically, PARC service consumers present with various unique challenges and a broad spectrum of individual needs. PARC staff are appropriately skilled to meet these demands, which was reflected in an improvement in the areas of consumer needs being identified and addressed, and the service being personalized to meet consumer’s unique needs. During consumer engagement, PARC staff applied a strength-based approach to their interviewing style, to help the consumer focus on what they are already good at, and proceed from there. This approach resulted in consumers feeling well supported with developing their recovery plan and also being encouraged to self-manage their plans. However, in reflection on these results, PARC indicated that further enhancement of “goal identification support” may be achieved through (a) the involvement and co-facilitation of trainers and teachers with lived experience from the “Mind Recovery College” and (b) upskilling staff in SBA practice, as the consumer goal setting process is directly impacted by the quality of staff skills.

Advance planning and self-management was one of the fundamental principles that supported the recovery. As recovery is fundamentally a self-directed process, the consumer must have the authority to exercise choices and make decisions based on his or her recovery goals (31). In alignment with PARC’s philosophy, the support person is there as a facilitator to encourage the consumer to set themselves small and attainable goals. PARC staff were functioning well in this role of support person, working from a strengths-based approach and providing personalized services based on consumer needs. The data from the IRPs also indicated that goal setting and self-management of the plans was a key ingredient of the recovery process undertaken by consumers of the PARC Cairns service.

Prevention And Recovery Care is committed to a family-inclusive approach through staff and stakeholder education on issues relating to the support of people with mental illness. Staff feedback indicates a strong focus on including informal carers such as families and friends in consumer recovery plans. However, the reality proved to be different. The involvement of and access to informal carers presented a real challenge, which is corroborated by contemporary literature. The low rate of involvement of informal carers was possibly due to privacy issues and consumer choice of family involvement. Some IRPs required limited or no contact with family, dependent on consumer goals and needs. However, family members and informal carers were fully involved by PARC staff and routinely considered in all phases of the consumers’ stay. Nevertheless, a low take-up of responses in the 2016 SRI 2 may have been an indicator of several issues including families experiencing crisis due to the acute mental health needs of their family members, data collection methods that may have been inappropriate for Aboriginal and Torres Strait Islander and culturally and linguistically diverse families and carers, and possibly survey fatigue. PARC has identified this as an area to be addressed in their action plan.

There are some limitations to this study. Fluctuating numbers of participants and feedback in the 2015 and 2016 data do not allow for straight-up comparison. Misunderstanding in the term “informal carer fully involved” may have produced ambiguous results. The 3-month period for the analysis of IRPs was randomly selected and may not be a representative sample. Despite the admission of 45 consumers during the assessed 3 months, only 12 rated their progress. According to PARC staff, the 3-month period was particularly out of character compared with the overall response rates throughout the lifetime of the service. Future analysis might be more accurate if data of IRPs were equally spread out over a 12-month period as, for example, for 1 month every quarter of the year. Moreover, the low response rate did raise the question as to why the number of completed ratings was that small. In consultation with PARC staff, it became apparent that some consumers found it at times too hard to focus on the questions, let alone providing an answer or a score. In such cases, a feasible strategy to overcome this issue may be the employment of people with lived experience, to aid in the data collection.

These limitations notwithstanding, the study has shown the appropriateness of the overall evaluation framework for monitoring and improving the quality of a mental health recovery residential service. In collaboration with PARC services, we have successfully developed an evaluation framework, which has the potential to stimulate creativity in improving consumer outcomes and providing improved evidence of service effectiveness. Throughout all stages of the participatory research design, we continuously improved the framework, in consultations and email communication, by adjusting evaluation questions and methods until we were satisfied. Stakeholder’s input and participation in the process was vital and contributed to the richness of perceptions and expectations, the framework’s credibility, the sharing of power, and the clarification of roles and responsibilities (32). After completion of stakeholder engagement, the service logic was developed. The task was to describe the service; how it was intended to run; and the expected outcomes. The service logic intended to assist in gaining a clear understanding of the purpose of the service and how it fits into the larger context of public health. Furthermore, it contributed to fairness and accuracy of the evaluation; facilitated a balanced assessment of strengths and weaknesses; and aided stakeholders’ understanding of how service features fit together and relate to the big picture (32).

## Conclusion

The development of this evaluation framework has been conducted in collaboration with Cairns PARC, an established Adult Prevention and Recovery service in North Queensland, and researchers from James Cook University and Central Queensland University. Its credibility lies in its scientific participatory research approach, which invites stakeholders from all levels of interest in the service, to provide input throughout the development stage of the framework. Their participation allows for diverse views and experiences to aid the development of an evaluation framework that is focused on better outcomes for consumers and their carers, as well as an improved service that is based on evidence and knowledge.

## Ethics Statement

This study is a quality assurance activity and was granted an exemption from ethical clearance by the Far North Queensland Human Research Ethics Committee (HREC/15/QCH/108—1006 QA).

## Author Contributions

All listed authors contributed substantially to the design, draft, analysis, and critical revisions of the manuscript.

## Conflict of Interest Statement

EW, JJ, TW, and SM were all employed by Mind Australia Ltd. LB was employed by the University of Melbourne and funded by Mind Australia Ltd., as Mind’s principal research fellow she provided expert advice to the project and supported the implementation of the SRI 2. All other authors declare no competing interests. Mind Australia had no role in experimental design or analysis. They provided data and info for the report, but did not analyze or write any of it.

## Supplementary Material

Figure S1 Prevention And Recovery Care (PARC) services’ evaluation logic model.
