# Supplementary material for: Evaluation of a Residential Mental Health Recovery Service in North Queensland
Source: Front Public Health. 2018 May 2;6:123. doi: 10.3389/fpubh.2018.00123 (PMC5940734; doi:10.3389/fpubh.2018.00123)
Supplement: Figure S1 — Prevention And Recovery Care (PARC) services’ evaluation logic model. [file image_1.PDF]

PARC PHILOSOPHY

based on the five core principals of the recovery orientated practice model by Mind Australia

1. Supporting personal recovery and promoting well-being

2. Delivering services informed by evidence and consistent with a social model of health

3. Proactive and purposeful engagement to build trusting relationships

4. Ensuring the practice is sensitive to the need of families and cares

5. Working in partnership and collaborating with other organisations

WHAT WE DO/ PROVIDE

to ensure

the PARC philosophy is fulfilled

- Deliver recovery based services including a mix of clinical, psychosocial treatment and rehabilitation support, education and training; advocacy of independence and quality of life for people at a crucial point of crisis, relapse or recovery
- Provide least restrictive environment/ facilities to clients given their mental health service needs

- Adopted a recovery oriented culture within the service underpinned by appropriate values and the service model that reflected in the service guidelines, staff orientation manual, daily routine and actions

- Employ a collaborative process of engagement in the service, i.e. participatory approach in daily psychological recovery review between client - PARC clinician, client – PARC keyworker
- Allocate a keyworker to assist with orientation to the service and recovery plan

- Facilitate discharge from inpatient care
- Allocate a keyworker to assist with orientation to the service and recovery plans
- Provide consolidated community transition and treatment plans
- Established a commitment to continuous quality improvement (CQI) of the service
- Ensure that staff reflects cultural diversity of clients

- Established sustained partnerships local communities and organisations

so that the following OUTCOMES occur

EXAMPLES of SHORT-TERM OUTCOMES

Recovery support provided

- Provided safe and culturally appropriate recovery based care
- Increased active input and client/carer participation in the service and individual care planning
- Reduced unnecessary inpatient admissions
- Reduced avoidable re-admissions
- Increased awareness of mental health related issues
- Minimised trauma and disruption for clients and carers that may arise from a first episode or relapse of mental illness
- Reduced the burden of care experienced by carers
- Established partnerships

EXAMPLES of INTERMEDIATE OUTCOMES

Capacity to live well in the community

- Improved mental health outcomes of people with a severe mental illness, who become acutely unwell
- Increased knowledge, skills and confidence to recognise and manage life stressors in a healthy way
- Increased ability to participate in the community, employment, education and training
- Improved coordination between primary care and specialist mental health services in the community to enhance consumer choice and facilitate integrated service provision

EXAMPLES of LONG-TERM OUTCOMES

Mental health status

- Reduced negative impact of mental health
- Increased good mental health and wellbeing
- Collected an evidence base to evaluate the PARC service value
